# Sexual Dysfunctions, Post-Traumatic Stress Disorder, and Postpartum Hemorrhage : Trait Anxiety as a moderator factor

**DOI:** 10.1192/j.eurpsy.2026.12053

**Published:** 2026-07-31

**Authors:** N. Smaoui, A. Jedidi, S. Omri, N. Charfi, H. Hkim, R. Feki, I. Gassara, M. Maalej, M. Maalej, L. Zouari

**Affiliations:** 1 Psychiatry C; 2 Gynecology-Obstetrics Department, Hedi Chaker Hospital, Sfax, Tunisia

## Abstract

**Introduction:**

Severe postpartum hemorrhage (PPH) is not only a major obstetric emergency but also a source of long-term psychological and relational difficulties. Both post-traumatic stress disorder (PTSD) and sexual dysfunction are frequent complications in this context, yet their interplay and underlying vulnerability factors remain underexplored. Trait anxiety, as a stable personality characteristic, may act as a key moderator influencing these outcomes.

**Objectives:**

The objectives of this study were to evaluate sexual dyfunction and post-traumatic stress disorder (PTSD) in women who experienced severe postpartum hemorrhage (PPH), and to investigate the role of trait anxiety in this relationship.

**Methods:**

A cross-sectional, descriptive, and analytical study was conducted between January and December 2024 among 49 participants who presented with severe PPH. These women had delivered between 2017 and 2023 at the Gynecology-Obstetrics Department of Hedi Chaker University Hospital in Sfax.

Sexual function was assessed using the Female Sexual Function Index (FSFI), with a threshold score ≤ 26.55 defining sexual dysfunction. PTSD was assessed using the Post-Traumatic Stress Disorder Checklist for DSM-5 (PCL-5), with a diagnostic threshold set at a total score ≥ 31. Trait anxiety was assessed using the Spielberger Trait Anxiety Inventory (STAI-Y).

Statistical analysis was performed using SPSS-26 software.

**Results:**

The mean FSFI total score was 19.3 ± 8.15 (min = 0, max = 34.5), with a prevalence of sexual dysfunction in 79.6% (n=39) of participants.

The mean PCL-5 score was 31.81 ± 18.54 (min = 4, max = 71); 44.9% of participants (n = 22) met DSM-5 criteria for PTSD.

Trait anxiety was present in 51% of participants and showed a strong association with PTSD (p < 0.001); women with an anxious personality profile were significantly more likely to develop PTSD than non-anxious participants. Trait anxiety was also significantly associated with sexual dysfunction (p = 0.031) and remained independently associated after adjustment in multivariate analysis (adjusted OR = 5.75; 95% CI 1.08–30.72; p = 0.041), indicating that anxious personality traits contribute to impaired sexual function following severe PPH.

**Conclusions:**

Trait anxiety was a significant moderator of PTSD and sexual dysfunction risk after severe PPH. These findings highlight the importance of systematic screening for anxiety traits to identify women at higher risk and guide targeted interventions to improve both psychological health and sexual well-being.

**Disclosure of Interest:**

None Declared

